# Children with special needs and their access to rehabilitation services in the Philippines: A Q methodology study on perceived barriers by family members

**DOI:** 10.1002/puh2.79

**Published:** 2023-04-04

**Authors:** Irish Mae C. Chiu, Michael P. Sy, Myra D. P. Oruga, Sheila R. Bonito

**Affiliations:** ^1^ Faculty of Management and Development Studies University of the Philippines Open University Los Baños Laguna Philippines; ^2^ National Teacher Training Center for the Health Professions University of the Philippines Manila Philippines; ^3^ College of Nursing University of the Philippines Manila Philippines

**Keywords:** childhood disability, health beliefs, mixed method, Philippines, Q‐methodology, social determinants of health, special needs, systemic discrimination

## Abstract

**Background:**

Childhood disability remains a lowly prioritized and funded international health concern in the world today. Exacerbated by poverty, a lack of bureaucratic support, and societal discrimination, rehabilitation services are not readily accessible for Filipino families who happen to have a child with disability. This article intends to determine the perceived barriers of Filipino service users when it comes to accessing rehabilitation services for children with special needs in Cagayan de Oro City and to propose suggestions to improve the access to rehabilitation services from an international health perspective.

**Methods:**

The study utilized the four‐phased Q methodology, a mixed‐method research design with an exploratory sequential approach: (1) creating and validating the Q‐sort statements, (2) Q‐sorting administration, (3) factor analysis, and (4) interpreting factors.

**Results:**

After going through the four phases of Q methodology, the following factors, called “viewpoints” emerged: systemic discrimination based on differences in culture and ethnicity (Viewpoint 1), socioeconomic factors such as affordability and accessibility (Viewpoint 2), and predisposed health beliefs and lack of trust to health professionals (Viewpoint 3).

**Discussion:**

Barriers to accessing rehabilitation services in the Philippines go beyond the lack of individual resources. These perceived barriers can be mitigated by employing participatory and collaborative approaches in developing rehabilitation programs for children and their families, viable strategies such as integrating telehealth in the rehabilitation process, and cultural competence in recognizing folkloric beliefs as a way to build trust toward health‐care professionals.

**Conclusion:**

This article determined contextualized barriers when accessing health and rehabilitation services based on service users themselves, which in turn hopes to promote inclusive, justice‐oriented, and culturally focused rehabilitation services underpinned by international health principles.

## Graphical abstract

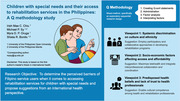

## INTRODUCTION

Children with disability require specific health‐care services such as rehabilitation and therapy services to address their condition due to impairments and developmental delays. Rehabilitation is a term that includes a wide range of interventions to address limitations in terms of activity and participation obstacles in addition to personal and environmental factors that influence daily functioning [1]. Therapy services are part of rehabilitation that aims to restore and compensate for the loss of function and prevent the deterioration in the functioning of all areas in a person's life. These services include patient education and advocacy to provide adequate support for the achievement of independence and participation in daily activities. To do that, a rehabilitation team is formed to support the patient. The team includes “occupational therapists, orthotists, physiotherapists, prosthetists, psychologists, rehabilitation and technical assistants, social workers, and speech therapists” [2]. To date, childhood disability remains a development goal for the health system but is still underprioritized [3] within global and international health agendas [4].

Out of 91 million Filipinos, 1.443 million are considered or are affected by disabled [5] as reported in 2010. Among the 17 administrative regions in the country, Region 4 has the highest number of persons with disabilities (PWDs) of ∼193,000, whereas the Cordillera Administrative Region (CAR) has the lowest number of PWDs of ∼26,000. Region 10 (Northern Mindanao), the setting for this study, was recorded to have 67,000 PWDs, whereas males comprised 50.9% in the 2010 census. In another national survey conducted by the Philippine Statistics Authority [6], results revealed that among the 10,240 respondents (who are 15 years old and above), 12% had “severe disability,” 47% had “moderate disability,” 22% had “mild disability,” and 19% had “no disability.”

In 2018, the World Health Organization [1] reported that barriers to accessing health services go beyond physical structures. This means that aside from the restrictive physical spaces, barriers can include the absence of basic assistive devices, antagonistic mentalities of individuals toward handicaps, lack of rehabilitative services, and non‐inclusive approaches. This study aimed to synthesize data gaps on the factors influencing the utilization of rehabilitation services by families with children with disability in Cagayan de Oro City (a city in Southern Philippines) to address the lack of research on the predicament of children with disabilities. The focus was to showcase what and how barriers are still present in a city with existing rehabilitation services. The lack of reliable data and under‐prioritization regarding disabilities [7] has hindered government agencies from understanding the extent to which PWDs are included and how they will be included in health and social services, including rehabilitation [8].

Although quantitative studies, albeit limited, had reported frequency analyses on disability data, a mixed‐method research design could potentially build on these quantitative data sets to provide more rigor and contextual understanding of complex and specific issues in disability research. The Q‐methodology, a mixed‐method research design, was chosen to include service users (or clients) as part of the study from start to end, gathering their perceptions and views and analyzing them to generate evidence that will inform practice and policies [9]. To do this, we formulated the following research objectives: (1) to determine the perceived barriers of service users (Filipino parents and family members) toward accessing rehabilitation services for children with special needs in Cagayan de Oro City; and (2) to propose suggestions on how to improve the access of rehabilitation services for children with special needs and their families from an international health perspective based on the qualitative comments that were obtained from the survey.

## METHODS

### Study design and participants

#### Q‐Methodology (QM)

QM is a mixed‐method research design with an exploratory sequential approach and was employed for this study. Particularly, we utilized the QM process conducted in another Filipino‐based study within the context of drug rehabilitation [10]. QM typically has four sequential steps: (1) creating and validating the Q‐sort statements, (2) Q‐sort administration, (3) factor analysis, and (4) interpreting factors. Among the four steps, steps 1 and 4 constitute the qualitative part, whereas step 3 constitutes the quantitative part. QM uses exploratory factor analysis and is not subjected to sample size estimation. Usually, the number of participants is smaller than the number of Q‐sort statements (QSS) in the Q‐set (i.e., the constellation of statements). Moreover, participants in QM studies should be carefully and purposively selected rather than randomized so that variability can be analyzed in the specific case or situation [11]. Given the estimated number of 33 QSS within the Q‐set for this study, the target sample size was 32. The participants were selected from the seven identified rehabilitation facilities that offered occupational, physical, and speech therapy services across Cagayan de Oro City. Inclusion criterion was primarily the participants’ willingness to participate in the study through online communication, social media, and/or phone call.

### Study variables and development of instrument

#### Q‐methodology step 1: creating and validating the Q‐sort statements

Each QSS contained statements regarding barriers that influence the utilization of rehabilitation services by families with children with a disability based on existing literature (see Table 1). A total of 33 QSSs (e.g., first QSS is coded as QSS1 and second QSS is coded as QSS2…) were initially generated, all of which were subjected to expert validation. Four experts were invited to validate the 33 QSS using Google Forms. These experts were purposively selected with either of these criteria must have at least 10 years of experience working as a rehabilitation service provider or have published a peer‐reviewed paper. Two (out of four) experts stated that QSS32 (lack of knowledge and negative attitude toward the rehabilitation of families among family members with children with disability) was similar to QSS 13 resulting in merging the two QSS. Minor amendments were also done to other QSSs to improve the readability of the QSS based on experts’ feedback. The final set of QSS was composed of 32 QSS, all of which were able to capture the constellation of factors that influence rehabilitation service utilization for children with disability and their families. Before administering the Q‐sorting, the final set of QSS was piloted on a sample of five participants. These pilot participants provided feedback to improve the statements, check the ease of the task, identify the average time for task completion, and other general suggestions about the QM process.

**TABLE 1:** Final set of Q‐sort statements (QSSs) each depicting a barriers that influence the utilization of rehabilitation services based on existing literature (total of 32 QSS).

QSS	Statement	Reference
1	Expensive costs of rehabilitation and therapy services	WHO (2013); Henly and Adams (2018); Abdi et al. (2015)
2	Limited availability of rehabilitation services	WHO (2013), Adugna et al. (2020), Zuurmond et al. (2019)
3	Barriers in the physical space and environments	WHO (2013), WHO (2011), Adugna et al. (2020)
4	Lack of knowledge, competencies, and credentials (e.g., valid professional license to practice) of health‐care providers	WHO (2013), Abdi et al. (2015), Kuwana (2014)
5	Persons with disabilities are reportedly being denied access to health care due to a lack of financial resources	WHO (2013)
6	Communication barriers such as lack of knowledge or familiarity with sign language or other adapted communication devices	Shakespeare et al. (2018), WHO (2011)
7	Attitudinal barriers include presuming low expectations of PWDs that keep people from appreciating and experiencing the full potential of PWDs	WHO (2011)
8	Negative attitudes include stigmatizing and discriminating against PWDs	Shakespeare et al. (2018), Adugna et al. (2020), Abdi et al. (2015)
9	Institutional barriers, such as laws, policies, systemic practices, or strategies, which intentionally or unintentionally discriminate against PWDs	WHO (2011)
10	Policy barriers such as denying PWDs access to certain programs and services, including workplaces, health care, and educational institutions	CDC (2019)
11	Barriers in accessing durable medical equipment and assistive devices (including expensive cost to maintain and repair these equipment and devices)	CDC (2019), Henly and Adams (2018), Kuwana (2014)
12	Transportation barriers include inaccessible or inconvenient transportation and distance from the rehabilitation center	CDC (2019), WHO (2011), Adugna et al. (2020), Abdi et al. (2015)
13	Insufficient knowledge, awareness, and understanding of the needs of PWDs among other groups in the community	Jacobs et al. (2011), Adugna et al. (2020)
14	Lack of coordination and collaboration among health‐care professionals	Jacobs et al. (2011)
15	Lack of training and financing for health‐care professionals	Jacobs et al. (2011)
16	Rehabilitation staff members who are not approachable and show poor interpersonal skills	Jacobs et al. (2011)
17	Complicated billing systems related to PhilHealth reimbursements (e.g., applying for reimbursements, securing requirements, and waiting for payments)	Jacobs et al. (2011)
18	Poor self‐esteem of clients, which makes it more difficult to access health‐care and rehabilitation services	Jacobs et al. (2011)
19	Insufficient knowledge about the referral process	Zuurmond et al. (2019)
20	The delay in referral to respective health specialists due to long queues and difficult scheduling	Jacobs et al. (2011)
21	Caregivers who lack trust in service providers	Jacobs et al. (2011), Zuurmond et al. (2019)
22	Inconsistency in terms of attendance of the health‐care service providers due to tardiness and absenteeism	Jacobs et al. (2011)
23	Limited opening hours of health‐care establishments that make it inconvenient for caregivers who are working (e.g., no services provided on Sundays when caregivers of PWDs are most available)	Jacobs et al. (2011)
24	Cultural and ethnic discrimination such as misconceptions and social constructions about the causes of disabilities	George et al. (2018), Adugna et al. (2020)
25	Insufficient policies and standards wherein the policy design does not always take into consideration the needs of PWDs	WHO (2011)
26	Superstitious beliefs and prejudices that constitute barriers to education, employment, health care, and social participation	WHO (2011)
27	Financing allocated to implementing policies and plans to support PWDs are insufficient	WHO (2011)
28	Lack of consultation and involvement wherein PWDs are not included from decision‐making in important matters affecting their lives	WHO (2011)
29	Lack of relevant data on disability and evidence‐based programs, more specifically, data that is locality specific (usually international data or data from urban areas)	WHO (2011)
30	Lack of privacy (e.g., photos and personal information of PWDs are being posted on social media without consent)	Adugna et al. (2020)
31	Weather conditions, disaster, pandemic, and climate change	Adugna et al. (2020), Kuwana (2014)
32	Factors such as socioeconomic status and social support of family and friends	Mishra and Siddharth (2018)

Abbreviation: PWD, persons with disabilities.

### Data collection

#### Q‐methodology step 2: Q‐sorting administration

Although some studies administered Q‐sorting in a face‐to‐face arrangement, some authors argued that Q‐sorting can also be conducted online [12]. Considering the COVID‐19 restrictions affecting the conduct of primary research, an online Q‐sort self‐administration arrangement was employed and was expected to last for about 45–60 min. In this process, the participants were requested to rank the 32 QSS according to their perspective, opinion, or viewpoint. Called the “general sorting” process, they were asked to place each QSS in their respective columns ranging from −4 (least agree) to +4 (most agree). It was emphasized that the manner of sorting should be based on their opinion and prioritization of the factors that influence the utilization of rehabilitation services by families with children with disability. After all the cards were arranged on the sorting grid presented on the computer screen, the participants were reminded to ensure that no card was left unsorted. A comment box was provided where the participants could type their clarifications, opinions, and comments. After completing the Q‐sorting process, each participant would have produced one Q‐sort equivalent to one set of data.

### Data analysis

#### Q‐methodology step 3: factor analysis

Demographic data were encoded using Microsoft Word, whereas the Q‐sort data sets were encoded into Ken‐Q Analysis desktop edition (KADE) version 2.0 free software [13]. The correlation analysis compared the views of the participants to find similarities and differences among their respective opinions or viewpoints, whereas the factor analysis categorized correlated participants’ opinions or viewpoints based on their finalized Q‐sorts under different factors. Every Q‐sort was correlated with all the existing Q‐sorts, then their intercorrelation matrix was factor‐analyzed using principal component analysis with varimax rotation.

A factor loading (factor variable correlation) was determined for each Q‐sort, expressing the extent to which each Q‐sort is associated with the other. The number of factors in the final set depends on the variability across the Q‐sorts. In other words, the correlation analysis distinguished the viewpoints among participants to find similarities and differences, whereas the factor analysis categorized correlated participants’ viewpoints. Each participant's rank‐ordered sort of statements was converted into an array of numerical data, each of which was intercorrelated with the arrays of the other participants. The correlation matrix presented who among the participants sorted the statements in a similar fashion; the matrix was subjected to factor analysis to achieve groupings of data arrays that are highly correlated. This allowed for the determination of the factors that would represent clusters of participants having the same viewpoints [14]. Each identified factor would be referred to as a “viewpoint.”

#### Q‐methodology step 4: interpreting factors

Factor scores were standardized to reflect *z*‐scores for each factor that were created by ranking the largest positive to largest negative *z*‐scores [15]. In other words, when a respondent's factor loading exceeds a certain limit (i.e., *p* < 0.01), this is called a *defining variable* (or variable). The difference score refers to the magnitude of difference between a statement's score on any two factors that are required for it to be statistically significant. These quantitative data were compared to the demographic profile of the participants as well as their comments on the questionnaire and were subjected to interpretation via content analysis duly conducted by the first two authors. Content analysis is the “systematic, objective, quantitative analysis of message characteristics” and this type of analysis was used because this included quantitative analysis of qualitative data such as correlation matrix and factor analysis [16]. In case of unforeseen circumstances, detailed notes regarding the QM process were written for documentation. Given the multiple steps employed, a triangulation process was utilized to mitigate potential biases and increase the validity, reliability, and rigor of the research process.

##### Ethical considerations

This study has been approved by the UP Open University Institutional Research Ethics Committee with study code: UPOU MIH202103. All participants were oriented on all the steps of the QM to be undertaken before signing the informed consent. Those who consented to participate voluntarily were anonymized to ensure confidentiality and privacy from data collection toward the completion of the analyses.

## RESULTS

This section would first discuss the results of the factor analysis and the description of the distinguishing statements per group. After going through the four steps of QM, the following factors, called “viewpoints,” emerged: systemic discrimination based on differences in culture and ethnicity (Viewpoint 1), socioeconomic factors such as affordability and accessibility (Viewpoint 2), and predisposed health beliefs and lack of trust to health professionals (Viewpoint 3).

### Results of the factor analysis

Factor loadings show the degree of correlation between an individual Q‐sort and each factor score correlation as shown in Table 2. (See Tables 3, 4, 5 The 1st column consists of the 32 Q‐sorts collected (respondent code), the next 3 columns refer to demographic data (respondents’ age, income level, and educational attainment), and the last 3 columns show the factor loading across the 3 identified factors (viewpoints).

**TABLE 2:** Factor loadings based on the 32 Q‐sorts through principal component analysis with varimax rotation.

Respondent code	Age	Income level	Educational attainment	Factor 1	Factor 2	Factor 3
13	37	Upper middle income	Postgraduate	**0.7811**	0.0350	−0.1197
14	43	Lower middle income	Postgraduate	**0.7571**	0.0116	0.1626
21	51	Rich	Postgraduate	**0.6515**	0.0128	0.0961
23	52	Low income	Tertiary	**0.6258**	0.0958	−0.1743
2	29	Rich	Postgraduate	**0.6095**	−0.0744	0.5644
17	28	Middle class	Tertiary	**0.6035**	−0.2420	0.2766
20	22	Upper income	Tertiary	**0.5919**	0.1958	0.0436
4	34	Low income	Tertiary	**0.5894**	0.4337	0.2494
11	33	Rich	Tertiary	**0.5688**	0.5106	−0.1482
16	31	Middle middle class	Tertiary	**0.5474**	0.2501	0.3737
1	37	Poor	Postgraduate	**0.5176**	0.1472	−0.3527
22	26	Middle middle class	Secondary	**0.4334**	0.0799	0.2948
18	36	Rich	Postgraduate	**0.4303**	0.2901	0.2348
15	34	Low income	Tertiary	**0.3594**	0.3190	0.1947
28	47	Low income	Tertiary	**0.3583**	0.0797	0.0407
9	49	Middle middle class	Tertiary	**0.3193**	0.0686	0.0871
25	35	Upper income	Tertiary	**0.0887**	0.0229	0.0207
30	42	Lower middle income	Postgraduate	0.1476	**0.8380**	0.0720
29	43	Upper income	Postgraduate	0.2519	**0.7995**	0.0603
24	31	Lower middle income	Tertiary	0.1040	**0.7879**	0.1367
27	44	Upper income	Tertiary	0.1846	**0.7700**	0.2313
5	44	Upper middle income	Tertiary	−0.0336	**0.7092**	0.3614
31	40	Middle middle class	Tertiary	0.0996	**0.4753**	−0.2596
6	33	Lower middle income	Tertiary	0.4493	**0.4548**	−0.1022
3	36	Lower middle income	Tertiary	0.2095	**0.4294**	−0.1771
26	33	Low income	Secondary	−0.0860	**0.3856**	−0.3048
8	39	Upper middle income	Tertiary	−0.0678	**0.3509**	−0.3410
19	44	Lower middle income	Tertiary	0.0387	0.1179	**−0.6562**
10	41	Middle middle class	Postgraduate	0.3022	0.1846	**0.6296**
12	43	Low income	Tertiary	−0.1340	0.2726	**0.5364**
7	33	Lower middle income	Tertiary	0.0669	0.0851	**0.2450**
32	32	Poor	Tertiary	−0.0620	0.0497	**−0.1484**
**% Explained variance**				**17**	**15**	**9**
**Number of defining variables**				**17**	**10**	**5**

*Note*: The figures in bold are the number of defining variables per factor. It is also important to note that the rank is derived from the weighted composites, where the sign * indicates significance at *p* < 0.05.

**TABLE 3:** Distinguishing statements for Viewpoint 1.

QSS	Statement	Rank	*z*‐Score
20	The delay in referral to respective health specialists due to long queues and difficult scheduling	3	1.24
1	Expensive costs of rehabilitation and therapy services	3	1.17
24	Cultural and ethnic discriminations such as misconceptions and social constructions about the causes of disabilities	2	1.14*
2	Limited availability of rehabilitation services	2	1.06*
9	Institutional barriers, such as laws, policies, systemic practices, or strategies, which intentionally or unintentionally discriminate against PWDs	1	0.61*
6	Communication barriers such as lack of knowledge or familiarity with sign language or other adapted communication devices	1	0.33*
7	Attitudinal barriers include presuming low expectations of PWDs that keep people from appreciating and experiencing the full potential of PWDs	0	0.20
11	Barriers in accessing durable medical equipment and assistive devices (including expensive cost to maintain and repair these equipment and devices)	0	0.02
26	Superstitious beliefs and prejudices that constitute barriers to education, employment, health care, and social participation	−1	−0.02*
12	Transportation barriers include inaccessible or inconvenient transportation and distance from the rehabilitation center	−2	−0.90*
15	Lack of training and financing for health‐care professionals	−2	−1.02
30	Lack of privacy (e.g., photos and personal information of PWDs are being posted on social media without consent)	−3	−1.14*
21	Caregivers who lack trust in service providers	−3	−1.51*
16	Rehabilitation staff members who are not approachable and show poor interpersonal skills	−3	−1.59*
22	Inconsistency in terms of attendance of the health‐care service providers due to tardiness and absenteeism	−4	−1.78*

*Note*: Figures with asterisk (*) are significant at less than or equal to 0.05.

Abbreviations: PWD, persons with disabilities; QSS, Q‐sort statements.

**TABLE 4:** Distinguishing statements for Viewpoint 2.

Number	Statement	Rank	*z*‐Score
1	Expensive costs of rehabilitation and therapy services	4	1.99*
8	Negative attitudes include stigmatizing and discriminating against PWDs	3	1.43
3	Barriers in the physical space and environments	3	1.36*
7	Attitudinal barriers include presuming low expectations of PWDs that keep people from appreciating and experiencing the full potential of PWDs	2	0.87*
13	Insufficient knowledge, awareness, and understanding of the needs of PWDs among other groups in the community	2	0.65*
23	Limited opening hours of health‐care establishments that make it inconvenient for caregivers who are working (e.g., no services provided on Sundays when caregivers of PWDs are most available)	0	−0.01
15	Lack of training and financing for health‐care professionals	0	−0.36*
25	Insufficient policies and standards wherein the policy design does not always take into consideration the needs of PWDs	−1	−0.38*
16	Rehabilitation staff members who are not approachable and show poor interpersonal skills	−1	−0.65*
21	Caregivers who lack trust in service providers	−2	−0.74*
2	Limited availability of rehabilitation services	−2	−0.84*
4	Lack of knowledge, competencies, and credentials (e.g., valid professional license to practice) of health‐care providers	−3	−0.89*
22	Inconsistency in terms of attendance of the health‐care service providers due to tardiness and absenteeism	−3	−0.98*
18	Poor self‐esteem of clients that makes it more difficult to access health care and rehabilitation services	−3	−1.20*
24	Cultural and ethnic discriminations such as misconceptions and social constructions about the causes of disabilities	−4	−1.74*
26	Superstitious beliefs and prejudices that constitute barriers to education, employment, health care, and social participation	−4	−1.86*

*Note*: Figures with asterisk (*) are significant at less than or equal to 0.05.

Abbreviation: PWD, persons with disabilities.

**TABLE 5:** Distinguishing statements for Viewpoint 3.

Number	Statement	Rank	*z*‐Score
26	Superstitious beliefs and prejudices that constitute barriers to education, employment, health care, and social participation	4	1.61*
21	Caregivers who lack trust in service providers	3	1.18*
10	Policy barriers such as denying PWDs access to certain programs and services, including workplaces, health care, and educational institutions	3	0.98*
16	Rehabilitation staff members who are not approachable and show poor interpersonal skills	2	0.98*
19	Insufficient knowledge about the referral process	2	0.68
1	Expensive costs of rehabilitation and therapy services	1	0.56
24	Cultural and ethnic discriminations such as misconceptions and social constructions about the causes of disabilities	1	0.48*
22	Inconsistency in terms of attendance of the health‐care service providers due to tardiness and absenteeism	0	0.41*
27	Financing allocated to implementing policies and plans to support PWDs are insufficient	0	−0.04*
7	Attitudinal barriers include presuming low expectations of PWDs that keep people from appreciating and experiencing the full potential of PWDs	−1	−0.36
31	Weather conditions, disaster, pandemic, and climate change	−2	−1.19*
15	Lack of training and financing for health‐care professionals	−3	−1.54
2	Limited availability of rehabilitation services	−3	−1.56*
14	Lack of coordination and collaboration among health‐care professionals	−4	−1.93*

*Note*: Figures with asterisk (*) are significant at less than or equal to 0.05.

Abbreviation: PWD, persons with disabilities.

The numbers represent the factor loadings that are correlation coefficients indicating the extent to which each of the 32 Q‐sorts is similar or different to each of the four composite factor arrays. The three identified factors explain 41% of the total variance within the range data. The rows indicate the participants’ opinions, represented by numerical values as factor loading under each factor. A positive value signifies that the participant perceives with the viewpoints of others, whereas a negative value signifies that a participant disagrees with the viewpoint. For example, the opinions of Q‐sort 13 correlate highly with factor 1, whereas Q‐sort 31 correlates more with factor 2 than 3. The following values that were in bold are “flagged,” which means that they are the most defining sorts for each of the three factors. It is also important to consider that not all Q‐sorts were automatically flagged because some Q‐sorts were not initially flagged requiring manual flagging to avoid being excluded from the analysis [12].

#### Viewpoint 1: systemic discrimination based on differences in culture and ethnicity

Seventeen respondents (out of 32) shared this viewpoint. Their responses largely encapsulate how culture and ethnicity influence the utilization of rehabilitation services such as misconceptions and social constructions about the causes of disabilities (24, +2 [QSS 24, ranked +2]) and limited availability of rehabilitation services (2, +2). Moreover, the group believes that barriers can include laws, policies, systemic practices, or strategies that intentionally or unintentionally discriminate against PWDs (9, +1) as well as communication barriers such as lack of knowledge or familiarity with sign language or other adapted communication devices (6, +1). On the other hand, this group did not believe that the following are barriers: tardiness and absenteeism (22, −4), unapproachable staff (16, −3), lack of trust in service providers (21, −3), and lack of privacy (30, −3).

Participant 1 (37, poor, postgraduate) conveyed that “there is lack of knowledge and awareness about children with special needs.” Most of the participants proposed the need for proper information dissemination about therapy services and the referral process. Participant 2 (29, rich, postgraduate) expressed that “the first step for a family to seek for rehabilitation services is to recognize that there is an existing need for assistance due to the delay on the development of their child.” For parents to recognize these delays, they must have sufficient knowledge, awareness, and understanding of what attributes to expect in their child's growth and development. Raising awareness and promoting education to both parents and caregivers is always the best remedy to immediately address and support every child's need.

#### Viewpoint 2: socioeconomic factors such as affordability and accessibility

There were 15 respondents who shared this viewpoint. For them, barriers included the expensive costs of rehabilitation and therapy services (1, +4 [QSS 1, ranked +4]), physical space, and environmental barriers (3, +3). They also believed that barriers include the attitudinal stigma such as presuming low expectations of PWDs (7, +2) as well as insufficient knowledge, awareness, and understanding of the needs of PWDs among other groups in the community (13, +2). However, the group disagrees with the following statements: superstitious beliefs and prejudices (26, −4), cultural and ethnic discrimination (24, −4), poor self‐esteem of clients (18, −3), inconsistency in terms of attendance of the health‐care service providers (22, −3), and lack of knowledge, competencies, and credentials of health‐care providers (4, −3).

Participant 15 (34, low income, Tertiary) also reported that “PWDs who have nothing to spend on therapies are unable to access the assistance they need. Too costly to the point that regular wage earners couldn't afford the therapies.” Participant 20 (22, upper income, tertiary) also stated that “people in low socio‐economic status have difficulty accessing therapy services due to lack of funding from the government, thus remaining untreated or even undiagnosed.”

#### Viewpoint 3: predisposed health beliefs and lack of trust in health professionals

There were nine respondents who shared this group. They responded that the factors influencing the utilization of rehabilitation services are superstitious beliefs and prejudices (26, +4 [QSS 26, ranked +4]), caregivers who lack trust in service providers (21, +3), and policy barriers (10, +3). On the contrary, this group disagrees with the following statements: lack of coordination and collaboration among health‐care professionals (14, −4), limited availability of rehabilitation services (2, −3), and lack of training and financing for health‐care professionals (15, −3).

Participant 10 (41, middle class, postgraduate) reported that “superstition is still evident especially in secluded areas where people are not aware of the benefits for PWDs and others felt that having a disabled family member is *nakakahiya* (embarrassing).” Participant 4 (34, low income, tertiary) stated that “bullying is very common to PWDs because the community lacks knowledge with regards to the health condition. This should be properly addressed because it can greatly affect the well‐being of a person with a disability (physical or behavioral/intellectual disability).”

Five consensus statements were also identified. In other words, these barriers were not distinguishable in any of the groups (factors), which means that there is no significant difference between any factors. It fails to distinguish one factor from others because all factors give the same statement and the same score. These consensus statements were as follows:

- QSS 5: PWDs are reportedly being denied access to health care due to a lack of financial resources;
- QSS 17: Complicated billing systems related to PhilHealth reimbursements (e.g., applying for reimbursements, securing requirements, and waiting for payments);
- QSS 28: Lack of consultation and involvement wherein PWDs are not included in decision‐making in important matters affecting their lives;
- QSS 29: Lack of relevant data on disability and evidence‐based programs more specifically, data that is locality‐specific (usually international data or data from Urban areas);
- QSS 32: Factors such as socioeconomic status and social support of family and friends.

## DISCUSSION

Childhood disability is a development priority in the health system [3], specifically in the Philippines. Cieza et al. [4] asserted that childhood disability must be integrated into the global and international health agenda. Although rehabilitation services are already known to be invariable and of poor quality in low‐ and middle‐income countries (LMICs) in Asia such as the Philippines, societal, structural, and attitudinal barriers exacerbate this international health concern [17]. The lack of priority as well as the lack of structural and bureaucratic support for childhood rehabilitation [18] remain barriers to health, which we need to examine and address through international health research. To further situate our findings, we arranged our interpretations based on the identified viewpoints.

### Viewpoint 1: systemic discrimination based on differences in culture and ethnicity

In developing countries like the Philippines, children with disabilities and their families still undergo discrimination, which hinders them from enjoying their basic human rights [19]. UNICEF has created a digest in 2007 on promoting the rights of children with disabilities, which largely espouses the social model of disability [20]. This particular model rejects the long‐established idea that barriers for PWDs are primarily from physical impairments, rather barriers are largely caused by environmental factors, including discrimination.

Discrimination toward PWDs includes posing negative attitudes, such as underestimation, non‐inclusive programs and policies, deprivation of access to health‐care and educational resources [21], physical barriers to access home and community environments, and the determinants of poverty and injustices [22]. Systemic discrimination refers to the patterns of behavior, policies, or practices that become a structured part of any institution or organization that perpetuates disadvantages for those people from marginalized backgrounds, including PWDs.

For children with disabilities, such patterns can be described as ableism. Hehir [23] defined ableism as “the devaluation of disability” that “results in societal attitudes that uncritically assert that it is better for a child to walk than roll, speak than sign, read print than read Braille, spell independently than use a spell‐check, and hang out with nondisabled kids as opposed to other disabled kids.” By definition, ableism is the discrimination of people favoring able‐bodied people over those with disability. The proliferation of ableist attitudes and practices in health care, especially in occupational therapy [24], do not adhere to the tenets of the social model of disability. Viewpoint 1 opens clarifications on how barriers can be systemic and patterned. In other words, systemic discrimination, characterized by ableist patterns of practices and attitudes, can be more covert but more dangerous when it comes to marginalizing children with disabilities and their families. The uncritical practices anchored on the hegemonic biomedical model in rehabilitation services perpetuate these patterned barriers. These, then, must be replaced by the integration of participatory, inclusive, and justice‐orientated practices [24] when designing and implementing rehabilitation programs that can improve desired outcomes toward inclusivity in health care.

### Viewpoint 2: socioeconomic factors such as affordability and accessibility

Although Viewpoint 1 focuses on discrimination as a barrier, Viewpoint 2 emphasizes socioeconomic factors as a collective barrier to accessing rehabilitation services. In the Philippines, a staggering 31.4% of children live below the poverty line [25], and one out of four (1:4) people with a disability, which means 5.1 million Filipino children live with disability [26].

Viewpoint 2 affirms that therapy services are too expensive for the participants resulting in inaccessibility or invariable availability. Although there is already an insurance package for children with developmental disabilities [27], most families still spend out‐of‐pocket to avail of these therapy services. Although we do not have any statistics on the capacity of Filipino families to pay for therapy services, a recent qualitative study by Lasco and associates [28] ascertained that parents of Filipino children with disabilities financially struggle to provide for their child's medical and therapy needs. During the pre‐pandemic period, children with disabilities had to travel an average of 5–6 km to reach the nearest health facility [29, 30, 31], and during the pandemic, telemedicine and teletherapy posed barriers, such as internet connectivity issues, internet budget constraints, and digital literacy concerns [32, 33, 34, 35]. Due to the lack of access to opportunities, such as therapy sessions, schooling, and community participation, parents would naturally be led to believe that lowering their expectations for their children would be the best recourse. This situation is even exacerbated when chronic sorrow and hopelessness are felt by parents, especially mothers, when reminded about their child's limitations during therapy sessions and everyday occurrences [36].

Considering socioeconomic factors in health access suggests that health‐care professionals should integrate telehealth as part of the therapy process and not just an option or alternative. Moreover, rehabilitation professionals should also enforce interprofessional competencies such as ensuring that they coordinate the needs of potential clients to accurate and rightful services within the health, social welfare, and community systems as well as inform them about existing health programs, in light of the Universal Health Care Law, which could reduce health‐care costs.

### Viewpoint 3: predisposed health beliefs and lack of trust in health professionals

Superstitious beliefs and prejudices also constitute barriers to education, employment, health care, and social participation [2]. In the Philippine context, superstitious beliefs largely influence the perception of people toward medical conditions and remedies, which decrease trust toward health professionals, especially those residing in the rural regions [37]. Instead of seeking medical advice, some Filipinos in rural Philippines resort to traditional folk healers whom they believe will be the only source of remedy through enchanment [38]. The strong belief in the metaphysical and spiritual realms largely influences Filipino families’ health‐related decisions for a family member who is sick or may need treatment due to a disabling condition. In Taiwan, parents of children with autism resort to fortune‐telling to know about the etiology of their child's condition because the cost of regular therapies can be expensive; hence, going to the nearest faith healer can be more viable and budget‐friendly [39].

Although respect for culture is warranted and some herbal medicines are proven effective, folk healers sometimes use shamanistic rituals that are believed to be effective even if there is no proven scientific evidence for it. The scarcity of funds for health care needs in developing countries encourages people to resort to more affordable remedies that are more accessible in the community such as folk healers. The affordances brought by folk healers to local families may lessen their trust toward licensed health and medical professionals. Where occupational therapists are encouraged to understand how finding comfort in traditional healing is important for some patients/clients [40], such interventions can delay clinical diagnosis, treatment, and prognosis for children with disabilities.

#### Limitations of the study

This is a study that utilized a small sample size in one study location. This means that findings are not meant to generalize but rather meant to provide a systematic way of organizing multisourced data in examining the barriers to accessing rehabilitation services among Filipino children with disability.

## CONCLUSION

This study utilized Q‐methodology, a type of mixed‐method research design, and determined the perceived barriers of service users (Filipino parents and family members) when it comes to accessing rehabilitation services for children with special needs in Cagayan de Oro City. These barriers include (1) systemic discrimination based on differences in culture and ethnicity, (2) socioeconomic factors such as affordability and accessibility, and (3) predisposed health beliefs and lack of trust in health professionals.

Despite the steady increase of children with disability around the world, childhood disability remains less prioritized and less funded internationally and in the Philippines. Poverty proves to be one of the culprits that keep children with disability deprived rather than participative in daily living. The identified viewpoints confirmed that these barriers were supported by existing literature and also illuminated some often‐overlooked issues surrounding childhood disability and their health concerns. Viewpoint 1 suggests that systemic discrimination, characterized by ableist patterns of practices and attitudes, can be more covert but more dangerous when it comes to marginalizing children with disabilities and their families. This viewpoint then proposes participatory and justice‐oriented approaches when developing awareness programs and the reevaluation of rehabilitation services underpinned by determinants of the social model of disability. Viewpoint 2 suggests that socioeconomic factors define what is health and determine their access to health for people under the margins. Bringing health services, such as preventive, promotive, and tele‐rehabilitation programs, closer to home via employing community therapists at the grassroots level can be a viable solution, especially in rural Philippines. Viewpoint 3 suggests that folkloric health beliefs supersede modern‐day health‐care provisions. This viewpoint proposes to turn this barrier into an ingredient for intervention. In other words, this helps health providers and clients recognize folkloric and superstitious beliefs and use this openness to build mutual trust throughout the rehabilitation process.

Moving forward, findings from this study propose that mixed‐method approaches in international health research are possible and valuable. More than providing empirical data to explain present issues in childhood disability, studies like this that involve service users promote the creation of authentic information that would be needed in developing rehabilitation programs for Filipino children with additional needs and their families; therefore, challenging the top–down and authoritative approach and espousing a more inclusive, justice‐oriented, and culturally focused approach in program and policy development.

## AUTHOR CONTRIBUTIONS

*Conceptualization; data curation; formal analysis; writing original draft until final editing (lead)*: Irish Castro. *Conceptualization; co‐development of the methodology; supervision; cowriting from original draft until final editing*: Michael Sy. *Conceptualization; validation; and final editing*: Myra Oruga. *Conceptualization and supervision*: Shiela Bonito.

## CONFLICT OF INTEREST STATEMENT

The authors have no relevant financial or nonfinancial interests to disclose.

## Data Availability

The data that support the findings of this study are available on request from the corresponding author. The data are not publicly available due to privacy or ethical restrictions.
